# Cone‐beam computed tomography image guided therapy to evaluate lumpectomy cavity variation before and during breast radiotherapy

**DOI:** 10.1120/jacmp.v14i2.4243

**Published:** 2013-03-04

**Authors:** Minh Tam Truong, Ariel E. Hirsch, Nataliya Kovalchuk, Muhammad M. Qureshi, Antonio Damato, Bradley Schuller, Nectaria Vassilakis, Michael Stone, David Gierga, John Willins, Lisa A. Kachnic

**Affiliations:** ^1^ Department of Radiation Oncology Boston University School of Medicine Boston MA

**Keywords:** breast radiotherapy, seroma, lumpectomy cavity, surgical clips, cone‐beam computed tomography

## Abstract

The purpose of this study was to evaluate the rate of change (RoC) in the size of the lumpectomy cavity (LC) before and during breast radiotherapy (RT) using cone‐beam computed tomography (CBCT), relative to the initial LC volume at CT simulation (${\text{CTV}}_{\text{LC}}$) and timing from surgery. A prospective institutional review board‐approved study included 26 patients undergoing breast RT: 20 whole breast irradiation (WBI) patients and six partial breast irradiation (PBI) patients, with surgical clips outlining the LC. The patients underwent CT simulation (${\text{CT}}_{\text{sim}}$) followed by five CBCTs during RT, once daily for PBI and once weekly for WBI. The distance between surgical clips and their centroid (D) acted as a surrogate for LC size. The RoC of the LC size, defined as the percentage change of D between two scans divided by the time interval in days between the scans, was calculated before (${\text{CT}}_{\text{sim}}$ to CBCT1) and during RT (CBCT1 to CBCT5). The mean RoC of D for all patients before starting RT was −0.25%/day (range, −1.3 to 1.4) and for WBI patients during RT was −0.15%/day (range, −0.45 to 0.40). Stratified by median ${\text{CTV}}_{\text{LC}}$, the RoC before RT for large ${\text{CTV}}_{\text{LC}}$ group (≥25.7cc) was 15 times higher (−0.47%/day) than for small ${\text{CTV}}_{\text{LC}}$ group (<25.7 cc) (−0.03%/day), p=0.06. For patients undergoing ${\text{CT}}_{\text{sim}}$ < 42 days from surgery, the RoC before RT was −0.43%/day compared to −0.07%/day for patients undergoing ${\text{CT}}_{\text{sim}}\geq 42$ days from surgery, p=0.12. For breast cancer RT, the rate of change of the LC is affected by the initial cavity size and the timing from surgery. Resimulation closer to the time of boost treatment should be considered in patients who are initially simulated within six weeks of surgery and/or with large ${\text{CTV}}_{\text{LC}}$.

PACS number: 87.55.de

## I. INTRODUCTION

Defining the lumpectomy cavity (LC) in patients undergoing breast conserving surgery followed by adjuvant radiotherapy (RT) is important for patients receiving whole breast irradiation (WBI) followed by a boost to the tumor bed, as well as patients receiving partial breast irradiation (PBI), as randomized data have shown improved local control with the addition of a boost.^(^ ^1^ ^)^ Accurate identification of the LC clinical target volume (${\text{CTV}}_{\text{LC}}$) is essential in identifying breast tissue at highest risk for harboring microscopic disease to ensure tumor control while sparing the normal breast tissue.^(^ ^2^ ^,^ ^3^ ^)^ Computed tomography (CT) planning for PBI and WBI has become a standard practice, although defining the surgical cavity using CT alone can pose difficulties in its differentiation from normal breast tissue as the breast density varies among patients. Surgical clips placed during breast conserving surgery can aid visualization of the LC on radiographs and CT to help define the limits of the LC and improve the accuracy of defining the clinical target volumes for PBI and for the breast boost treatment in WBI.^(^ ^4^ ^–^ ^6^ ^)^ Variations in the LC during RT for WBI and PBI are not routinely integrated into the planning process and treatment for the majority of patients undergoing breast radiotherapy. Cone‐beam computed tomography (CBCT) is an image‐guided RT tool which could potentially be used to evaluate LC variations during RT when surgical clips are placed in the LC. CBCT can be used to identify patients at most risk for experiencing volume changes in the LC during RT which has important implications for treatment planning in defining the ${\text{CTV}}_{\text{LC}}$ and planning target volumes. In addition, CBCT can help determine the optimal timing for simulation of LC boost treatment and PBI so that the ${\text{CTV}}_{\text{LC}}$ drawn at simulation most accurately defines real‐time LC during RT.

The purpose of this study was to evaluate changes in the LC by investigating the rate of change (RoC) in size of the LC (with the distance (D) between the surgical clips and their centroid as a LC size surrogate) as a function of LC volume defined at CT simulation (${\text{CTV}}_{\text{LC}}$) and time interval between surgery and simulation CT (${\text{CT}}_{\text{sim}}$).

## II. MATERIALS AND METHODS

### A. Patient population

The study was conducted as a prospective single arm study with Institutional Review Board (IRB) approval. All patients signed written informed consent prior to entering the protocol. Twenty‐six patients, who were candidates for adjuvant WBI and PBI (Stage 0‐IIA breast cancer), with surgical clips outlining the LC, were enrolled in this study and treated with breast RT at our institution from 2007 to 2010. Twenty patients received WBI to 50 Gy in 25 fractions or 50.4 Gy in 28 fractions followed by the tumor bed boost of five or seven 2 Gy fractions. Six patients were treated with PBI at a dose of 40 Gy in 10 fractions twice a day (separated by 6 hours) on an IRB‐approved prospective PBI study. Full patient and tumor characteristics are detailed in Table 1.

**Table 1:** Select patient and tumor characteristics.

	*Median*		*Range*
Age at radiation therapy (years)	56		40–75
		*n (percent)*	
Race			
White		10 (38.5%)	
Black		9 (34.6%)	
Other		7 (26.9%)	
Cancer location			
Right breast		14 (53.8%)	
Left breast		12 (46.2%)	
Type of cancer			
Ductal Carcinoma In Situ (DCIS)		6 (23.1%)	
Invasive Ductal Carcinoma (IDC)		18 (69.2%)	
Mucinous Carcinoma of the Breast		1 (3.8%)	
Invasive Lobular Carcinoma (ILC)		1 (3.8%)	
Tumor stage			
Tis		6 (23.1%)	
T1		18 (69.2%)	
T2		2 (7.7%)	
Nodal stage			
N0		25 (96.2%)	
N1		1 (3.8%)	
Stage grouping			
0		6 (23.1%)	
I		17 (65.4%)	
II		3 (11.5%)	
Tumor grade			
I		9 (34.6%)	
II		13 (50.0%)	
III		4 (15.4%)	
Estrogen receptor (ER) status			
Positive		24 (92.3%)	
Negative		2 (7.7%)	
Progesterone receptor (PR) status			
Positive		18 (69.2%)	
Negative		8 (30.8%)	
Human epidermal growth factor receptor (HER2) status^a^			
Positive		4 (16.7%)	
Negative		20 (83.3%)	

a Not available for two patients.

### B. Imaging studies

All patients underwent CT simulation in supine position on a breast board (Bionix Max 2 TM, TorsoBoard Immobilization, Breast Board, Toledo, OH). Planning CT images (Brilliance CT Big Bore, Philips Medical Systems, Cleveland, OH) were acquired using 3 mm interslice thickness extending from the neck to at least 5 cm below the inframammary fold. The median time interval between the surgery and ${\text{CT}}_{\text{sim}}$ was 42 days (range, 20–300 days). To monitor the LC during treatment, patients underwent five CBCTs during RT (once weekly for WBI patients and once daily for PBI patients). CBCTs were acquired by the Varian On‐Board Imager (OBI) (Varian Medical Systems, Palo Alto, CA) versions 1.3 and 1.4, low‐dose thoracic mode, 512×512 matrix, interslice thickness of 2.5 mm. The median time interval between ${\text{CT}}_{\text{sim}}$ and CBCT1 for all patients was 15 days (range, 7–41 days). A total of 123 CBCTs were performed: 23 patients underwent five CBCTs, one patient underwent four CBCTs, and two patients underwent two CBCTs during breast RT.

### C. determination of lumpectomy cavity volume and size

The volume of excised breast tissue at surgery (${\text{SV}}_{\text{LC}}$) was estimated by calculating the volume of spheroid using specimen dimensions from pathology reports. ${\text{CTV}}_{\text{LC}}$ was contoured on ${\text{CT}}_{\text{sim}}$ by the treating radiation oncologist using information from the preoperative mammography, operative and pathology report, surgical clips, and postoperative changes seen on the ${\text{CT}}_{\text{sim}}$. A median of five clips (range, 3–12) were placed at surgery. The LC volume was computed by the Pinnacle treatment planning system software (Pinnacle versions 7.6c, 8.0m, Phillips Medical Systems, Andover, MA).

The mean centroid distance (D) in millimeters represented a distance between the surgical clips and their centroid:

(1) $$D=\frac{\sum_{i=1}^{N}\sqrt{{\left(x_{i}-\frac{\sum_{i=1}^{N}x_{i}}{N}\right)}^{2}+{\left(y_{i}-\frac{\sum_{i=1}^{N}y_{i}}{N}\right)}^{2}+{\left(z_{i}-\frac{\sum_{i=1}^{N}z_{i}}{N}\right)}^{2}}}{N}$$

where *N* is the number of clips, and $x_{i}$, $y_{i}$, and $z_{i}$ are the DICOM coordinates of the ith clip. This distance served as a surrogate for the LC size and was determined at ${\text{CT}}_{\text{sim}}$ and at each CBCT.

### D. determination of rate of change (roC) of the LC size

The RoC of the LC size was defined as the percent change of mean centroid distance D between two scans divided by the time interval in days between the scans:

(2) $$RoC=\frac{\left(\frac{\Delta D}{D}\right)}{\Delta T}\times 100$$

where *D* is the mean centroid distance in mm, Δ*D* is the difference in *D* between two scans in mm, and Δ*T* is the time in days between two scans.

The RoC before RT was represented by the change in the D between ${\text{CT}}_{\text{sim}}$ and CBCT1 for all (n=26) patients. For WBI patients, the RoC during RT was represented by the changes in the D between CBCT1 and CBCT5. Seventeen patients (excluding six PBI patients and three WBI patients with missing CBCTs) were included in the analysis to monitor the RoC during RT.

### E. Statistical analysis

Descriptive statistics were calculated for patient, tumor, and treatment‐related characteristics. Based on the median ${\text{CTV}}_{\text{LC}}$ (median=25.7 cc, range 4.9–230 cc), patients were divided into two groups: small ${\text{CTV}}_{\text{LC}}$ (<25.7 cc) or large ${\text{CTV}}_{\text{LC}}$ (≥25.7cc). Similarly, the median days from surgery to ${\text{CT}}_{\text{sim}}$ (median=42 days, range 20–300 days) were used as a cutoff to classify patients into an early (< 42 days) and late (≥42days) group. The RoC of the LC size was compared among groups using independent sample t‐tests. The Pearson correlation coefficient was used to test the correlation between various clinical factors. All statistical tests were two‐sided, and a probability value of less than 0.05 was considered statistically significant. The SAS System (Release 9.1, SAS Institute Inc, Cary, NC) was used to perform all statistical analyses.

## III. RESULTS

### A. Lumpectomy cavity measurements

The median ${\text{SV}}_{\text{LC}}$ and ${\text{CTV}}_{\text{LC}}$ were 27.0 cc (range, 4.2–121.4) and 25.7 cc (range, 4.9–230.6), respectively. The lumpectomy volume at ${\text{CT}}_{\text{sim}}$ correlated significantly with volume of excised breast tissue at surgery (Pearson correlation coefficient r=0.55, p=0.004).

The distribution of D at ${\text{CT}}_{\text{sim}}$ and each CBCT is presented in Table 2. During the time interval from ${\text{CT}}_{\text{sim}}$ to CBCT1 (median 15 days), LC size decreased in 17 patients (65%). For WBI and PBI patients, the median time interval between CBCT1 and CBCT5 was 28 and 4 days, respectively. Among the subset of available WBI patients (n=17), the LC decreased in 16 patients (94%) during RT. The percent reduction in median D between ${\text{CT}}_{\text{sim}}$ and CBCT1 was 13.7% and between CBCT1 and CBCT5 was 6.3% for WBI patients.

**Table 2:** Treatment characteristics of breast cancer patients treated with whole and partial breast irradiation therapy.

	*n*	*Median*	*Range*
${\text{SV}}_{\text{LC}}$ volume of excised breast tissue at surgery, cc	26	27.0	4.2–121.4
${\text{CTV}}_{\text{LC}}$ volume at ${\text{CT}}_{\text{sim}}$, cc	26	25.7	4.9–230.6
Number of clips placed at surgery	26	5	3–12
Time between surgery and ${\text{CT}}_{\text{sim}}$, days	26	42	20–300
Time between ${\text{CT}}_{\text{sim}}$ and CBCT1, days	26	15	7–41
Mean centroid distance (*D*), mm		*All subjects*	
${\text{CT}}_{\text{sim}}$	26	18.3	6.6–35.2
CBCT1	26	15.8	7.2–35.8
CBCT2	26	15.6	7.1–36.6
CBCT3	24	15.2	6.9–37.5
CBCT4	24	15.4	6.6–37.0
CBCT5	23	14.9	6.4–35.4
		*patients only*	
CBCT1	20	15.8	7.2–35.8
CBCT2	20	15.6	7.1–36.6
CBCT3	18	15.2	6.9–37.5
CBCT4	18	15.4	6.6–37.0
CBCT5	17	14.9	6.4–35.4
		*PBI patients only*	
CBCT1	6	15.0	11.5–22.3
CBCT2	6	14.6	11.5–22.9
CBCT3	6	14.8	12.2–22.3
CBCT4	6	14.8	10.9–22.9
CBCT5	6	14.7	10.7–22.7
		*WBI patients with complete CBCTs*	
CBCT1	17	15.9	7.2–35.8
CBCT2	17	15.9	7.1–36.6
CBCT3	17	15.0	6.9–37.5
CBCT4	17	15.4	6.6–37.0
CBCT5	17	14.9	6.4–35.4
CBCT5	17	14.9	6.4–35.4

n= number of patients; LC= lumpectomy cavity; CT= computed tomography scan; CBCT= Cone Beam CT; WBI= whole breast irradiation; PBI= partial breast irradiation.

### B. roC of the LC size

The RoC before (${\text{CT}}_{\text{sim}}$ to CBCT1) and immediately after RT (CBCT1 to CBCT2) was −0.248%/day and −0.259%/day, respectively. The overall RoC during RT was −0.151%/day. The distribution of the RoC is presented in Table 3.

**Table 3:** Rate of change (RoC) of the lumpectomy cavity size.

			*Rate of change (RoC), %/day*		
	*N*	*Median Days*	*Min*	*Mean*	*Max*
			*All subjects*		
CTsim to CBCT1	26	15	−1.297	−0.248	1.383
			*WBI patients only*		
CBCT1 to CBCT2	20	7	−1.472	−0.259	0.646
CBCT2 to CBCT3	18	7	−3.394	−0.234	0.893
CBCT3 to CBCT4	18	7	−1.842	−0.174	0.540
CBCT4 to CBCT5	17	7	−1.113	−0.075	1.170
CBCT1 to CBCT5	17	28	−0.449	−0.151	0.396

### C. RoC before and during RT stratified by lumpectomy cavity volume at ${\text{CT}}_{\text{sim}}$ (${\text{CTV}}_{\text{LC}}$)

**Figure 1:**
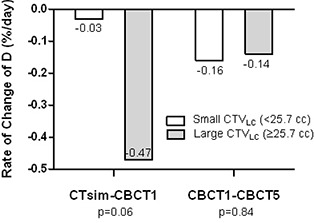
Rate of change of mean centroid distance before and during RT, stratified by initial LC volume at CT simulation (${\text{CTV}}_{\text{LC}}$).

Before RT, the mean RoC for large ${\text{CTV}}_{\text{LC}}$ group (≥25.7cc) was 15 times higher (−0.47%/day) than that for the small group (<25.7 cc) (−0.03%/day), p=0.06. During RT, mean RoC for the two groups was −0.16%/day for small ${\text{CTV}}_{\text{LC}}$ group and −0.14%/day for large group, p=0.84. Six PBI patients and three WBI patients who did not have five CBCTs during their treatment were excluded from the analysis of RoC during RT. The results are plotted in Fig. 1.

### D. RoC before and during RT stratified by time interval between surgery and ${\text{Ct}}_{\text{sim}}$

**Figure 2:**
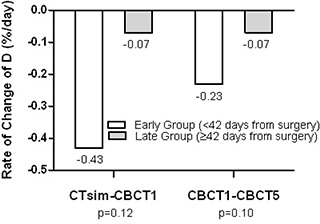
Rate of change of mean centroid distance before and during RT, stratified by time interval from surgery to CT simulation.

Before RT, the mean RoC for early group (${\text{CT}}_{\text{sim}}$ < 42 days) was six times higher (−0.43%/day) than that for late group (${\text{CT}}_{\text{sim}}\geq 42$) (−0.07%/day), p=0.12. During RT, mean RoC decreased by a factor of 2 for the early group (−0.23%/day) and remained the same for the late group (−0.07%/day), p=0.10. The results are shown in Fig. 2.

## E. RoC before RT stratified by both time from surgery and ${\text{CTV}}_{\text{LC}}$

**Figure 3:**
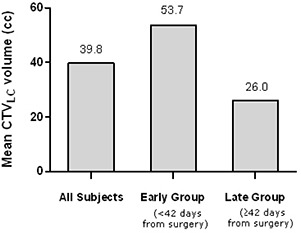
Effect of time from surgery on initial LC volume at CT simulation (${\text{CTV}}_{\text{LC}}$).

The early/large group (time to ${\text{CT}}_{\text{sim}}$ < 42 days and large cavity volume of ≥25.7cc at ${\text{CT}}_{\text{sim}}$) associated with quickest RoC in LC size (decrease of −0.48%/day) was followed by a late/large group (decrease of −0.40%/day), suggesting that size was a more important determinant of the pretreatment change compared to time. The RoC in early/small group was −0.14%/day, while late/small group had the slowest rate (−0.01%/day). We were unable to perform similar analysis on RoC during RT due to small number of patients available for analysis. When correlating the time interval between surgery and ${\text{CT}}_{\text{sim}}$ with lumpectomy cavity volume at ${\text{CT}}_{\text{sim}}$ (${\text{CTV}}_{\text{LC}}$), the mean ${\text{CTV}}_{\text{LC}}$ volume for early group (53.7 cc) was twice as large as ${\text{CTV}}_{\text{LC}}$ volume for late group (26.0 cc), p=0.12 (Fig. 3).

## IV. DISCUSSION

In this study, we prospectively analyzed serial CBCT scans to monitor change in the LC defined by surgical clips before and during RT. With the mean centroid distance between clips and their centroid serving as a surrogate for LC size, we demonstrated that the RoC of the LC size was affected by both the size of ${\text{CTV}}_{\text{LC}}$ and the time period from surgery to ${\text{CT}}_{\text{sim}}$. The RoC was the highest in patients simulated within six weeks from surgery. After six weeks of surgery, no difference was noted in the RoC before and during RT. The RoC before RT was found to be higher in patients with a larger ${\text{CTV}}_{\text{LC}}$ prior to starting RT, whereas ${\text{CTV}}_{\text{LC}}$ volume did not affect the RoC during RT. A comparison of this study with select previous studies^(^ ^2^ ^,^ ^5^ ^,^ ^7^ ^–^ ^19^ ^)^ is presented in Table 4.

**Table 4:** Summary of select studies on variation in postsurgical lumpectomy cavity before and during breast radiotherapy.

					*Change in lumpectomy cavity volume*			
*First Author, year*	*N*	*Treatment*	*Imaging Technique*	*Surgical Clips*	*Before RT* ^a^	*During RT* ^a^	*Correlated with time from surgery*	*Correlated with initial LC volume*
Vicini, 2003^7^	18	PBI	CT	Yes	NR	Yes	NR	NR
Weed, 2004^5^	28	17 PBI, 11 WBI	CT	Yes	NR	Yes^b^	Ye s ^c^	NR
Jacobson, 2006^8^	20	WBI	CT	No	NR	Yes	NR	Yes
Oh, 2006^9^	30	WBI	CT	Yes	NR	Yes	Yes^c^	Ye s
Kader, 2008^2^	205	PBI	CT	No	Yes	NR	Yes^c^	NR
Hurkmans, 2009^10^	10	WBI	CT	Yes	NR	Yes	Yes^c^	NR
Harris, 2009^11^	11	WBI	CT, PI	Yes	Yes	Yes	NR	NR
Hepel, 2009^12^	30	WBI	CT	Yes^d^	NR	Yes	Yes^c^	Ye s
Prendergast, 2009^13^	36	30 WBI, 6 PBI	CT	Yes	Yes	Yes	Yes	Yes
Tersteeg, 2009^14^	77	WBI	CT	Yes^d^	NR	Yes	NR	Yes
Sharma, 2009^15^	24	WBI	CT	Yes^d^	NR	Yes	Yes^c^	NR
Flannery, 2009^16^	43	WBI	CT	Yes^d^	NR	Yes	Yes	Yes
Yang, 2010^17^	102	WBI	CT, CBCT^|e^	No	NR	Yes	Yes^c^	Ye s
Yang, 2010^18^	19	WBI	CT, CBCT	No	NR	Yes	NR	NR
Kim, 2010^19^	13	PBI	CT	Yes^d^	Yes	NR	NR	NR
Present Study	26	20 WBI, 6 PBI	CT, CBCT	Yes	Yes	Yes	Yes	Yes

a Before RT refers to period of LC change assessment where a second imaging scan was performed before the start of RT while during RT refers to time period where a second imaging scan was performed during or at end of RT.

b Second CT was performed just before, during or day of completion of treatment.

c An inverse relationship was observed between time from surgery and change in LC volume.

d Clips were used when available.

e 10 patients underwent multiple CBCT scans during RT and data was analyzed to assess correlation between CT and CBCT scans for seroma contouring.

N=number of patients; RT=Radiotherapy; LC=lumpectomy cavity; PBI=partial breast irradiation; CT=computed tomography scan; NR=not reported; WBI=whole breast irradiation; PI=Portal imaging; CBCT=Cone Beam CT.

The majority of the studies of postsurgical LC variations compared volumes of a baseline ${\text{CTV}}_{\text{LC}}$ contoured on ${\text{CT}}_{\text{sim}}$ with LC volume contoured on one CT acquired during RT^(^ ^7^ ^–^ ^9^ ^,^ ^11^ ^,^ ^12^ ^,^ ^14^ ^–^ ^17^ ^)^ at varying time points in a retrospective fashion. In this study, as patients were entered prospectively, time points of CBCT measurement were performed consistently between patients. Interestingly, when we excluded patients with small ${\text{CTV}}_{\text{LC}}$ < 15 cc at ${\text{CT}}_{\text{sim}}$, using a similar criterion in a study by Jacobson et al.,^(^ ^8^ ^)^ the RoC before RT was greater (−0.334%/day) in patients with large ${\text{CTV}}_{\text{LC}}$ compared to all patients (−0.248%/day), highlighting effect of initial ${\text{CTV}}_{\text{LC}}$ on RoC. Other studies have shown a mean reduction in ${\text{CTV}}_{\text{LC}}$ ranging from 22.5% to 77% between initial ${\text{CT}}_{\text{sim}}$ and boost ${\text{CT}}_{\text{sim}}$.^(^ ^8^ ^,^ ^9^ ^,^ ^12^ ^,^ ^14^ ^–^ ^16^ ^,^ ^19^ ^)^

In the Topolnjak et al. study,^(^ ^20^ ^)^ CBCT and digital reconstructed radiographs (DRR) were used to compare differences in setup error. DRR‐based setup errors were found to be smaller than the CBCT‐based setup errors, although DRR registration may underestimate the setup error based on bony anatomy compared to CBCT. However, evaluation of the LC was not studied. While CBCT has been used to verify treatment set up and may be particularly useful for patients undergoing PBI,^(^ ^21^ ^)^ Yang et al.^(^ ^17^ ^,^ ^18^ ^)^ compared CT with CBCT during breast RT to evaluate the LC and identified mean seroma reductions of 54% to 64% using CT and CBCT, although the objective of their study was to determine consistency of seroma cavity contouring between CT and CBCT scans (which were shown to be in good agreement), as well as interobserver concordance.

Prendergast et al.^(^ ^13^ ^)^ studied LC volume changes both before and after commencing RT in 36 patients undergoing WBI (n=30) and PBI (n=6). CT scans were performed shortly after surgery, at ${\text{CT}}_{\text{sim}}$ and before the start of the treatment boost. Before commencing RT, the ${\text{CTV}}_{\text{LC}}$ decreased by a median of 49.9% (−2.1%/day) compared to a median reduction of 44.6% (−0.95%/day) during RT. Interestingly, in a subset of patients who experienced a delay in the start of RT, the RoC before RT was −0.40%/day which was considerably lower than the overall median reduction of ‐2.1%. We also noted a higher reduction rate in LC size of −0.248%/day before RT compared to −0.151%/day during RT.

A majority of the studies reported an inverse association between the time from surgery to ${\text{CT}}_{\text{sim}}$ and change in LC before^(^ ^2^ ^)^ and during RT,^(^ ^5^ ^,^ ^9^ ^,^ ^10^ ^,^ ^12^ ^,^ ^15^ ^,^ ^17^ ^)^ with a longer time from surgery associated with a smaller LC change. The results for initial ${\text{CTV}}_{\text{LC}}$ and change in ${\text{CTV}}_{\text{LC}}$ were mixed, with some studies reporting larger volume reduction over time with a larger initial ${\text{CTV}}_{\text{LC}}$,^(^ ^12^ ^,^ ^14^ ^,^ ^16^ ^,^ ^17^ ^)^ while others reported no correlation.^(^ ^8^ ^,^ ^9^ ^,^ ^13^ ^)^

Kader et al.^(^ ^2^ ^)^ found that the optimal time to perform a ${\text{CT}}_{\text{sim}}$ for PBI patients was within 7–8 weeks from surgery, whereas, if ${\text{CT}}_{\text{sim}}$ was performed after eight weeks from surgery, then difficulties in identifying the LC arose. Our study showed that the LC may be subject to greater variation in size during RT if ${\text{CT}}_{\text{sim}}$ was performed <six weeks (42 days) from surgery, especially for large initial ${\text{CTV}}_{\text{LC}}$ ≥25.7cc (Fig. 2). The application of serial CBCT during treatment can identify LC variations which would require replanning. Finding the optimum time to replan a patient for the boost treatment after WBI appears to be a function of timing from surgery. These findings suggest that for patients undergoing initial ${\text{CT}}_{\text{sim}}$ within six weeks of surgery and with large LC, should be reevaluated during WBI with repeat ${\text{CT}}_{\text{sim}}$ acquired as close as possible to the timing of the start of the boost. This could result in a change of the LC boost volume and boost treatment technique, which may have implications for the overall breast volume treated and long‐term cosmetic outcome. Limitations of using CBCT for defining the LC include the inferior soft‐tissue resolution of CBCT compared to ${\text{CT}}_{\text{sim}}$ that poses uncertainty in ${\text{CTV}}_{\text{LC}}$ contouring.

Limitation of the study lies in the use of the mean distance between LC clips and their centroid as a sole surrogate for LC size and, thus, the RoC measurements reflected a single‐dimensional rather than a volumetric evaluation of LC change. The rationale in using D for LC evaluation rather than LC contour‐based volume was due to poor anatomic resolution of the seroma on CBCT compared to CT, which would introduce potential inter‐ and intraobserver variability in ${\text{CTV}}_{\text{LC}}$ definition. Finally, assessment of the RoC during treatment in our PBI population over a four‐to‐five day period requires greater patient numbers to establish a consistent pattern of LC changes during PBI, although the RoC during PBI treatment appears to be minimal. In contrast, LC variations in the WBI patient population can be significantly influenced by the time from surgery to ${\text{CT}}_{\text{sim}}$, especially for patients with large ${\text{CTV}}_{\text{LC}}$, and the LC may continue to contract during RT; this may be important in terms of timing of the ${\text{CT}}_{\text{sim}}$ for planning the boost treatment following WBI.

## V. CONCLUSIONS

The current study demonstrates that lumpectomy cavities can change in size before and during breast RT, especially if the first simulation is performed less than six weeks from the time of surgery and for patients with large LC. For these patients, resimulation closer to the time of boost planning to assess reduction in the size of the LC should be performed, to improve localization of the boost volume for WBI. Based on these results, we plan on evaluating the role of repeat CT acquired four weeks into WBI for breast boost planning in patients who were initially ineligible for electron boost based on the CT for WBI.
